# A 3D‐Bioprinted Functional Module Based on Decellularized Extracellular Matrix Bioink for Periodontal Regeneration

**DOI:** 10.1002/advs.202205041

**Published:** 2022-12-14

**Authors:** Xueting Yang, Yue Ma, Xiuting Wang, Shengmeng Yuan, Fangjun Huo, Genzheng Yi, Jingyi Zhang, Bo Yang, Weidong Tian

**Affiliations:** ^1^ State Key Laboratory of Oral Diseases National Engineering Laboratory for Oral Regenerative Medicine Engineering Research Center of Oral Translational Medicine Ministry of Education Department of Oral and Maxillofacial Surgery West China Hospital of Stomatology Sichuan University Chengdu 610041 P. R. China; ^2^ State Key Laboratory of Oral Diseases National Engineering Laboratory for Oral Regenerative Medicine Engineering Research Center of Oral Translational Medicine Ministry of Education West China Hospital of Stomatology Sichuan University Chengdu 610041 P. R. China; ^3^ Chengdu Shiliankangjian Biotechnology Co., Ltd. Chengdu 610041 P. R. China

**Keywords:** 3D bioprinting, bioink, decellularized extracellular matrix (dECM), dental follicle, periodontal regeneration

## Abstract

Poor fiber orientation and mismatched bone–ligament interface fusion have plagued the regeneration of periodontal defects by cell‐based scaffolds. A 3D bioprinted biomimetic periodontal module is designed with high architectural integrity using a methacrylate gelatin/decellularized extracellular matrix (GelMA/dECM) cell‐laden bioink. The module presents favorable mechanical properties and orientation guidance by high‐precision topographical cues and provides a biochemical environment conducive to regulating encapsulated cell behavior. The dECM features robust immunomodulatory activity, reducing the release of proinflammatory factors by M1 macrophages and decreasing local inflammation in Sprague Dawley rats. In a clinically relevant critical‐size periodontal defect model, the bioprinted module significantly enhances the regeneration of hybrid periodontal tissues in beagles, especially the anchoring structures of the bone–ligament interface, well‐aligned periodontal fibers, and highly mineralized alveolar bone. This demonstrates the effectiveness and feasibility of 3D bioprinting combined with a dental follicle‐specific dECM bioink for periodontium regeneration, providing new avenues for future clinical practice.

## Graphical abstract

A biomimetic and functional periodontal module is developed using the methacrylate gelatin/decellularized extracellular matrix bioink by employing a 3D bioprinting approach. The module simulates natural anatomical complexity and biochemical microenvironment, and exhibits strong biological induction activity. Well‐aligned periodontal fibers, highly mineralized alveolar bone, as well as satisfactory bone–ligament interface integration are achieved after 3 months of implantation in vivo.

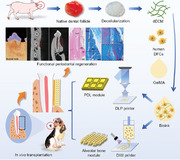

## 1 Introduction

Periodontal disease, characterized by progressive alveolar bone destruction and loss of periodontium, is the leading cause of tooth loss in adults worldwide.^[^ ^1^ ^]^ The ultimate goal of periodontal treatment is to regenerate tooth‐supporting tissue and form new attachments. Techniques such as bone grafting, guided tissue regeneration, and growth factor addition have been used in clinical practice to regenerate periodontal tissues, but limited therapeutic effects have been achieved due to restricted indications, poor attachment of long junctional epithelium, and collapse of the guided membrane.^[^ ^2^, ^3^, ^4^ ^]^ The reconstruction of periodontal tissues still remains highly challenging.

Periodontal tissue has an ingenious anatomical structure, including hard tissues such as alveolar bone and cementum as well as the well‐aligned soft tissue periodontal ligament (PDL), which is anchored between two hard tissues at a certain angle to maintain the stability of the teeth and facilitate chewing.^[^ ^5^, ^6^ ^]^ The key to achieving functional periodontal regeneration lies in the sandwich structure of the alveolar bone–PDL–cementum complex, and the appropriate PDL‐fiber orientation and functional bone–ligament interfaces become the principal barrier.^[^ ^7^, ^8^ ^]^ Currently, cell‐based tissue engineering techniques have achieved great progress in the field of periodontal regeneration. Jiang et al. fabricated an aligned nanofiber‐embedded scaffold, which provided topographic cues and subsequently guided the oriented regeneration of the periodontium.^[^ ^9^ ^]^ Our previous study constructed an aligned, electrospun poly(lactic‐co‐glycolic acid)/gelatin sheet and found that it could promote the formation of cellular cementum and the oriented arrangement of periodontal fibers in vivo.^[^ ^10^ ^]^ However, current regeneration strategies often fail to engineer hard/soft tissue interfaces. The major drawbacks of these strategies might be the imprecise control of material patterns and the spatial orientation of stem cells, which greatly limits the integrated regeneration of the periodontal sandwich composite.

As an innovative biofabrication strategy, the cell‐based 3D bioprinting technique has provided a new means to bioengineer various tissues and organs, especially the construction of bionic periodontal complexes with oriented alignment.^[^ ^11^, ^12^, ^13^, ^14^ ^]^ Combined with computer‐aided design, the printer could control the arrangement and distribution of materials at the micron scale to recapitulate human tissue complexity.^[^ ^15^, ^16^ ^]^ Bioprinted stem cells can be accurately located while giving the biomaterial the desired shape and architecture.^[^ ^17^, ^18^ ^]^ Therefore, bioprinting may represent significant progress over the conventional construction method and allow the fabrication of a biomimetic and functional periodontal sandwich composite.

To bioengineer periodontal tissues, a bioprinting scaffolding system should not only have topographical cues for aligning the PDL but also provide an inductive microenvironment to ensure efficient odontogenic and osteogenic differentiation. In recent years, decellularized extracellular matrix (dECM) bioink has attracted widespread attention.^[^ ^19^, ^20^ ^]^ Studies have demonstrated the benefits of tissue‐specific dECM bioinks, such as recreating the intrinsic ECM complexity of native tissues, mimicking ECM composition or resident cytokines, and enhancing stem cell differentiation into a specific lineage.^[^ ^21^, ^22^, ^23^ ^]^ 3D bioprinting based on dECM bioink has made great progress in heart, muscle, cartilage, skin and kidney regeneration.^[^ ^22^, ^23^, ^24^ ^]^ In this case, the combination of micron‐scale topographical cues with biochemical ECM components in 3D bioprinting might also bring new prospects for functional periodontal regeneration. However, due to the heavy demand of tissue sources to produce dECM bioink, the PDL, with a thickness of only 300 µm, does not have adequate material to serve as a source.^[^ ^8^ ^]^ As the precursor of periodontal tissue, dental follicles eventually develop into periodontal structures during tissue differentiation and maturation, providing a beneficial periodontal microenvironment.^[^ ^25^, ^26^ ^]^ Moreover, dental follicle tissue is more integrated and larger in size, and there is more tissue to serve as a source for bioink. Therefore, dental follicle‐derived dECM bioink may be an appropriate candidate for periodontal regeneration based on 3D bioprinting.

**Figure 1:**
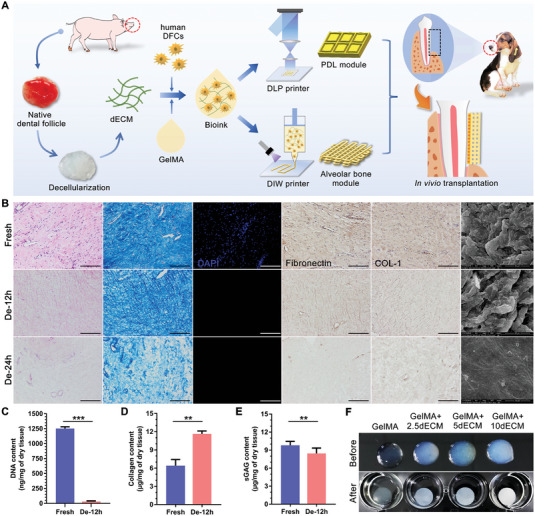
Fabrication and characterization of the porcine dental follicle‐derived dECM. A) Schematic overview of the 3D bioprinting of periodontal modules with GelMA/dECM bioink encapsulating human DFCs for periodontal regeneration. B) Histological comparison of dECM and fresh dental follicle tissues with H&E, Masson's trichrome, DAPI, and ECM protein staining and SEM analysis. C) Quantitative detection of DNA removal (***p = 3 × 10^−6^, *n* = 4 independent replicates from four samples), D) collagen retention (**p = 0.0012, *n* = 3 independent replicates from three samples) and E) sGAG content (**p = 0.0016, *n* = 3 independent replicates from three samples) in the dECM. F) Macroscopic appearance of bioinks with different dECM concentrations before and after crosslinking. Scale bars = 100 µm. (Two‐sided comparison, error bars represent standard deviation, *p < 0.05, **p < 0.01 and ***p < 0.001).

In this work, a biomimetic and functional periodontal module comprising PDL and alveolar bone was developed through 3D bioprinting (**Figure** **1**A). The called functional module is a kind of tissue module with specific function of the target organ based on stem cells and tissue engineering technology. According to the histological structure characteristics of the target organ, the module is assembled and spliced in a specific spatial arrangement, which finally realize the regeneration of the tissue and organ.

Here, porcine dental follicle‐derived dECM was added to methacrylate gelatin (GelMA), forming GelMA/dECM cell‐laden bioink, which showed excellent mechanical properties, printability as well as biocompatibility. Referring to topographical cues of the directional arrangement of natural periodontal fibers, a high‐precision PDL module was constructed by digital light projection (DLP) bio‐printer and succeeded in guiding the spatial orientation of encapsulated dental follicle cells (DFCs). Then, the direct ink writing (DIW) bio‐printer was used to form an alveolar bone module with decussate grid structure. The addition of dECM provided a favorable biochemical microenvironment, which successfully improved the viability of encapsulated DFCs as well as fibrogenesis and osteogenic differentiation. In a canine critical‐size periodontal defect model, the custom‐sized PDL module was first attached to the root surface, with alveolar bone module covered subsequently to form the intact periodontal functional module. After a 3‐month healing cascade, the integrated module incorporating dECM effectively restored the functional bone–ligament interfaces and the orientation and maturity of PDL fibers, with satisfactory height and thickness recovery of alveolar bone. Collectively, this study not only provides a new design and manufacturing method for the periodontal complex to solve the bottleneck problem of PDL orientation and hard/soft interface reconstruction but also develops a porcine dental follicle‐derived dECM bioink that is feasible for periodontal regeneration with more integrated microenvironmental clues.

## 2 Results

### 2.1 The GelMA/dECM Bioink Possessed Good Mechanical Properties, Printability, and Biocompatibility

Appropriate decellularization treatment time was the first and most important step in obtaining porcine dental follicle‐derived dECM. H&E and Masson's staining revealed the absence of cellular components in both decellularized tissues, but the fibrous extracellular matrix was seriously damaged in the 24 h treatment group (Figure 1B). Fibronectin and collagen immunostaining also showed various degrees of ECM protein reduction after decellularization, while the 12 h treatment group retained relatively more protein (Figure 1B). Scanning electron microscope (SEM) images showed that the 12 h treatment group had a surface structure and porosity similar to those of fresh tissue. In the 24 h treatment group, only the nanofiber mesh structure remained, indicating destruction of ECM components (Figure 1B). Therefore, treatment with Triton X100 and DNase I for 12 h was suitable for the decellularization of porcine‐derived dental follicle tissue. To further assess the remaining components in the dECM, a quantitative analysis of the major biological composition was performed. The DNA content was reduced by more than 96% (Figure 1C), and this change was also confirmed by DAPI staining (Figure 1B). The collagen (Figure 1D) and sulfated GAG (sGAG) content (Figure 1E) of the native and decellularized tissue indicated the retention of the active ingredients. Afterward, different concentrations of the obtained dECM were dissolved and mixed with GelMA to obtain the hydrogel. The transparency of the liquid hybrid hydrogel gradually decreased with increasing dECM concentration. After photocrosslinking, the high concentration of the GelMA/dECM ink became opaque milky white, while the pure GelMA remained colorless and transparent (Figure 1F).

**Figure 2:**
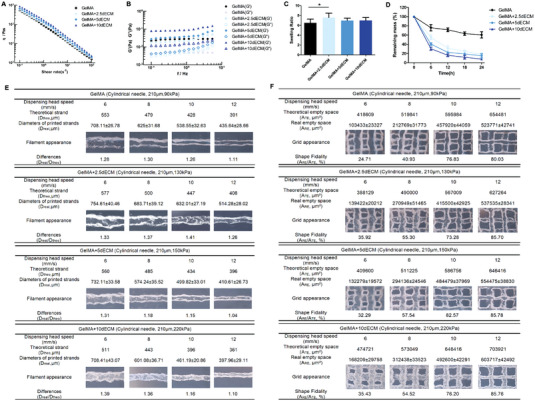
The GelMA/dECM bioink possessed good mechanical properties and printability. A) Viscosity and B) dynamic modulus (*G*′: storage modulus, *G*′′: loss modulus) measured at 25 °C. C) Swelling ratio in PBS (*p = 0.013). D) The degradation curve of pure GelMA and GelMA with dECM under the action of the enzyme. E) Filament diameters affected by printing speed for each condition. F) Value of shape fidelity under different printing speeds for each group. *n* = 3 independent replicates from three samples. (Two‐sided comparison, error bars represent standard deviation, *p < 0.05, **p < 0.01 and ***p < 0.001).

Rheology is a useful tool for determining the mechanical properties of bioinks for extrusion‐based 3D bioprinting.^[^ ^27^ ^]^ Our results showed that the viscosity (**Figure** **2**A) and dynamic modulus (Figure 2B) of the hydrogels gradually increased with increasing dECM concentration. The shear viscosities of the four inks all decreased linearly in the measured shear rate range, indicating that the addition of dECM did not affect the shear thinning behavior of the GelMA hydrogel (Figure 2A). The storage and loss moduli were associated with the elastic and viscous behavior of the bioinks, respectively.^[^ ^11^ ^]^ In the entire range of frequencies, the storage moduli (*G*′) were higher than the loss modulus (*G*′′) in the four bioinks (Figure 2B). This indicated that the GelMA/dECM bioinks could resist distortion and exhibited the elastic nature of a robust hydrogel network.^[^ ^11^ ^]^ The swelling ratio of GelMA did not significantly change by adding dECM (Figure 2C). In addition, the GelMA/dECM bioink degraded more rapidly than the pure GelMA hydrogel, suggesting that more protein and organic components were contained in the dECM (Figure 2D). It was also observed that a higher dECM concentration led to faster degradation.

3D printing requires hydrogels with good extrusive printability. We compared the minimum extrusion pressures (could be extruded from the nozzle but could not be printed continuously) and stable extrusion pressures (could be extruded from the nozzle and printed continuously) of the four groups and found that the pressure values increased with dECM concentration (Table S1, Supporting Information). However, the greater the extrusion pressure is, the greater the shear stress and the greater the cell damage.^[^ ^28^, ^29^ ^]^ With increasing printing speed, the diameter of the printed filaments decreased for each of the four hydrogels (Figure 2E). The filament diameters were all larger than the theoretical filament diameters. This was mainly due to the spreading of the extruded bioink when deposited on the platform. The *D*_real_/*D*_theo_ value of the GelMA+5dECM group was the lowest (from 1.04 to 1.31), which may be due to the appropriate viscosity and extrusion pressure to endow it with better plasticity.^[^ ^30^ ^]^ The printed grid depicted in Figure 2F indicated that the shape fidelity for each hydrogel increased with increasing printing speed. The GelMA+5dECM group still maintained the highest shape fidelity at dispensing head speeds of 8, 10, and 12 mm s^−1^, followed by the GelMA+10dECM group.

**Figure 3:**
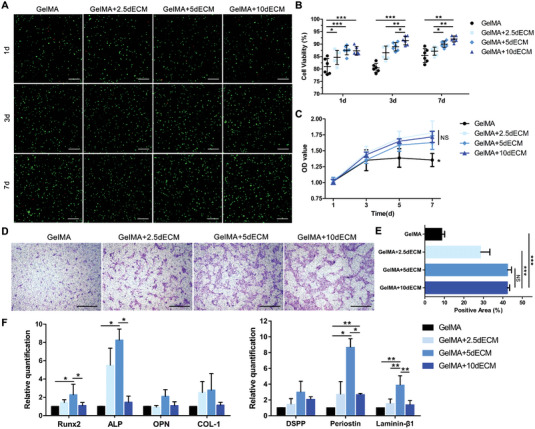
The GelMA/dECM bioink provided a favorable inductive microenvironment for DFCs. A) Live/dead staining and B) cell viability analysis of DFCs in the GelMA/dECM bioink for 7 days (*p = 0.0188, ***p = 0.0002, ***p = 0.0003, *p = 0.0421, **p = 0.0014, ***p = 7.55 × 10^−9^, *p = 0.0427, **p = 0.0012, **p = 0.0063, *n* = 6 fields from three independent samples). C) Cell proliferation of DFCs in GelMA/dECM hydrogels determined via a CCK‐8 quantitative analysis after 7 days of culture (*p = 0.0297, NS = 0.1788, 0.5635, 0.4091). D) Evaluation of the chemotactic activities of the GelMA and GelMA/dECM bioink diluent on DFCs by transwell assay. E) Quantitative statistics of the positive area of DFCs that migrated to the opposite side of the membrane (NS = 0.9416, ***p = 6.2098 × 10^−7^, ***p = 1.383 × 10^−12^, *n* = 6 fields from three independent experiments). F) Real‐time PCR detection of osteogenic (Runx2, ALP, OPN, COL‐1) and odontogenic (DSPP, periostin, laminin‐*β*1) gene expression in DFCs cultured in the four hydrogels for 7 days (left: *p = 0.0351, *p = 0.0478, *p = 0.0267, *p = 0.0109; right: *p = 0.0203, *p = 0.0297, **p = 0.0098, **p = 0.0012, **p = 0.0042, **p = 0.0028, *n* = 3 independent replicates from three experiments). Scale bars = 200 µm. (Two‐sided comparison, error bars represent standard deviation, *p < 0.05, **p < 0.01 and ***p < 0.001).

The effect of dECM on the biological behavior of stem cells in the hydrogel was further investigated. In this study, the human DFCs was selected as the seeding cells. On the one hand, we aimed to understand whether xenograft materials and cells elicit appreciable immune responses in vitro and in vivo. On the other hand, it was also desirable to verify whether the transplanted human DFCs are involved in the formation of new periodontal tissues in beagles. Live/dead staining showed the homogeneous distribution of DFCs throughout the hydrogels and the maintenance of high cell viability in all groups (**Figure** **3**A). The addition of dECM improved the survival rate of DFCs in hydrogels, and more living cells were observed in the GelMA+5dECM and GelMA+10dECM groups at days 1, 3, and 7 (Figure 3B). Growth curves of DFCs were analyzed using the CCK‐8 assay and showed that, in particular, the absorbance in each dECM group was higher than that in the GelMA group at days 5 and 7, indicating the capacity of dECM to promote cell proliferation (Figure 3C). However, no significant difference was found between the three GelMA/dECM groups. After 12 h of culture in the wells of a Transwell plate, cells that migrated to the lower surface of the membrane were stained with 1% crystal violet (Figure 3D). The addition of dECM to the GelMA hydrogel significantly improved cell mobility. The GelMA+5dECM and GelMA+10dECM groups exhibited the strongest migration‐promoting ability, followed by the 2.5dECM group (Figure 3E). After being cultured in hydrogel for 7 days, significantly higher expression of the osteogenic markers Runx2 and ALP and the odontogenic markers periostin and laminin‐*β*1 in the DFCs were found in the GelMA+5dECM group than in the other groups (Figure 3F). This might be attributed to the optimal modulus and porosity of the GelMA+5dECM bioink for DFCs. Numerous groups have used polyacrylamide gels with tunable elastic moduli to show that substrate stiffness affects various cell processes, including proliferation, migration, and stem cell differentiation.^[^ ^31^ ^]^ Under the dual effects of the suitable viscoelasticity of the bioink and the biomimetic ECM microenvironment, the DFCs encapsulated in GelMA+5dECM hydrogel showed excellent differentiation potential, which was superior to pure GelMA bioink.

Considering the mechanical and biological properties of the bioink comprehensively, we finally chose GelMA+5dECM as the optimal concentration for subsequent experiments.

### 2.2 GelMA/dECM Hydrogels Significantly Promoted Fibrogenesis and Osteogenic Differentiation after 3D Bioprinting

**Figure 4:**
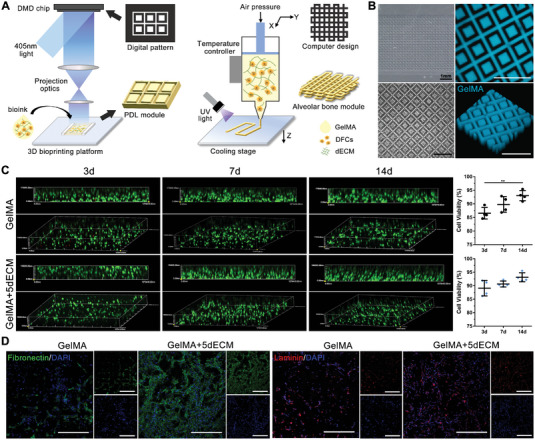
The 3D bioprinted PDL module promoted fibrogenesis and directional alignment. A) Schematic of the DLP‐based (left) and DIW‐based (right) 3D printers. B) Macroscopic and microscopic images of the printed PDL module observed under stereoscopic, inverted and confocal microscopes. C) 3D reconstruction images of live/dead staining of DFCs in the PDL module and the cell viability analysis after 3, 7, and 14 days (**p = 0.0035, *n* = 4 fields from four independent samples). D) IF staining of fibronectin and laminin in the GelMA and GelMA+5dECM groups on day 7. Scale bars = 500 µm. (Two‐sided comparison, error bars represent standard deviation, *p < 0.05, **p < 0.01 and ***p < 0.001).

Based on the 3D bioprinting technique, we created periodontal soft and hard tissues, including the PDL module using DLP bioprinter and the alveolar bone module using DIW bioprinter, to achieve architectural integrity and biological function (**Figure** **4**A). The stereoscopic and confocal microscopy images showed that the printed PDL module achieved lattice‐type and anatomically equivalent constructs along a predesigned geometry (Figure 4B). These constructs exhibited high shape fidelity, structural integrity, and geometrical stability. Under an inverted microscope, circular DFCs were scattered throughout the module (Figure 4B). Additionally, we performed 3D reconstruction of live/dead staining images and found significant differences between the two groups with the same structure (Figure 4C). The cytoskeleton of DFCs in the GelMA+5dECM group extended into a spindle shape along the long axis of the hydrogel column at 7 days, and then arranged in bundles along the *z*‐axis at 14 days. However, the DFCs in the GelMA group was mostly spherical at 7 days of culture and gradually aggregated into cell masses at 14 days, without obvious directionality. This discrepancy might be because the dECM possessed a stronger migration‐promoting ability, making DFCs less prone to aggregation. The cell viability of both groups was over 85% at day 3 and reached 92% at 14 days after the 3D printing process. This result indicated that light irradiation did not affect encapsulated cell survival within the PDL module and that the designed structure provided ample space for nutrient exchange. Additionally, immunofluorescence (IF) staining of the fibroblastic proteins fibronectin and laminin showed that the addition of dECM improved the fibroblastic differentiation ability of DFCs, which was crucial for PDL regeneration (Figure 4D).

**Figure 5:**
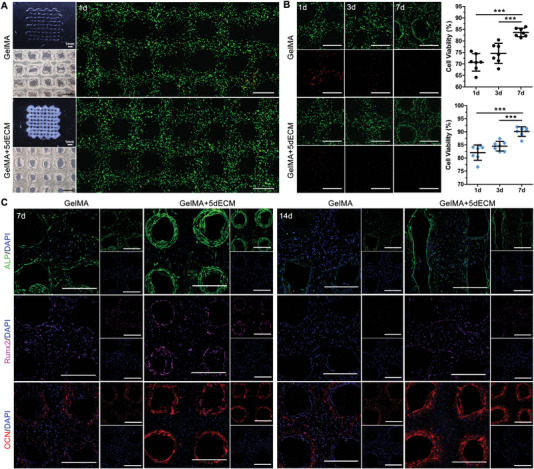
The 3D bioprinted alveolar bone module promoted osteogenic differentiation. A) Macroscopic and microscopic images of the printed alveolar bone module observed under stereoscopic, inverted and confocal microscopes. B) Live/dead staining and cell viability analysis of DFCs in the alveolar bone module for 7 days (***p = 2 × 10^−6^, ***p = 1.22 × 10^−4^, ***p = 3 × 10^−6^, ***p = 2.09 × 10^−4^, *n* = 7 fields from four independent samples). C) IF staining of the osteogenic‐related proteins ALP, Runx2 and OCN in the GelMA and GelMA+5dECM groups on days 7 and 14. Scale bars = 500 µm. (Two‐sided comparison, error bars represent standard deviation, *p < 0.05, **p < 0.01 and ***p < 0.001).

For the alveolar bone module, we chose the classic spatial grid structure in bone tissue engineering (**Figure** **5**A). The average diameter of the printed strands and spacings was 410.61 ± 26.73 µm. The images of live/dead staining on day 1 showed an evident lattice structure, and the DFCs were confined to the printed strands (Figure 5A). After 7 days of in vitro culture, the live/dead staining results showed that DFCs gradually stretched and migrated to the edge of the strand for better nutrient exchange (Figure 5B). One day after the bioprinting process, the cell viability of DFCs was only 70.67 ± 3.81% in the GelMA group, which might be due to the shear stress exerted by the extrusion of the cell‐laden bioink from the printing nozzle.^[^ ^32^ ^]^ This situation was improved after adding dECM, where the cell viability on day 1 reached ≈82.06 ± 2.92%. This result indicated that the dECM provided a favorable microenvironment for cell survival and reduced the adverse effects of shear stress and light irradiation on DFCs, consistent with the results shown in Figure 3B. After 7 days, the GelMA+5dECM group still showed higher viability (90.11 ± 1.83%) of encapsulated cells than the GelMA group (83.71 ± 1.84%). The expression of osteogenic‐related proteins in both alveolar bone modules was also examined by IF staining (Figure 5C). Higher amounts of the early osteogenesis markers ALP and Runx2 and the late marker OCN produced by encapsulated DFCs were found in the GelMA+5dECM group compared with the GelMA group at all experimental time points. The positive expressions of proteins were mostly located at the edge of strands. In addition, with the prolongation of in vitro culture time, the expression of early osteogenic markers in the alveolar bone module gradually decreased at 14 days; hence, we considered 7 days of in vitro culture to be a suitable time for subsequent orthotopic transplantation.

### 2.3 The dECM Bioink Featured Strong Immunomodulatory Activity In Vitro and In Vivo

**Figure 6:**
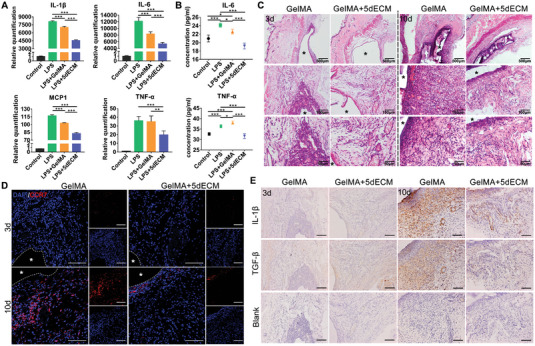
The dECM bioink suppressed M1 macrophage activation in vitro and in vivo. A) Real‐time PCR detection of M1 (IL‐1*β*, IL‐6, MCP1 and TNF‐*α*) marker gene expression in Raw264.7 cells cocultured with LPS and hydrogels for 3 h (IL‐1*β*: ***p = 1.4 × 10^−5^, ***p = 1.3424 × 10^−9^, ***p = 2.3871 × 10^−8^; IL‐6: ***p = 1.53 × 10^−4^, ***p = 6 × 10^−6^, ***p = 2.8 × 10^−5^; MCP1: ***p = 2 × 10^−6^, ***p = 2.5152 × 10^−9^, ***p = 1.8271 × 10^−7^; TNF‐*α*: ***p = 7.99 × 10^−4^, **p = 0.0063). B) ELISA analysis of the concentrations of the inflammatory factors IL‐6 and TNF‐*α* in the supernatant (IL‐6: *p = 0.0144, *p = 0.0205, ***p = 3.27 × 10^−4^, ***p = 1.87 × 10^−4^, ***p = 1.4 × 10^−5^; TNF‐*α*: *p = 0.0352, ***p = 6.84 × 10^−4^, ***p = 4.9 × 10^−5^, ***p = 1.3 × 10^−4^, ***p = 1.4 × 10^−5^). C) H&E staining of the bioprinted module at 3 and 10 days after subcutaneous transplantation (asterisk: implanted module). D) Immunostaining of CCR7 in the subcutaneous tissues at 3 and 10 days after surgery (white asterisk: implanted module). E) IHC evaluation of the inflammatory factors IL‐1*β*, IL‐17 and TGF‐*β* at 3 and 10 days after subcutaneous transplantation. Scale bars = 100 µm. *n* = 3 biologically independent replicates (Two‐sided comparison, error bars represent standard deviation, *p < 0.05, **p < 0.01 and ***p < 0.001).

To evaluate the immunomodulatory function of the dECM bioinks, lipopolysaccharide (LPS)‐treated macrophages were used to mimic the inflammatory environment, and the expression of inflammation‐related genes in the treated cells after coculture with the diluted inks was analyzed by PCR (**Figure** **6**A). The results showed that the addition of LPS successfully induced polarization of M1 macrophages and created an inflammatory environment, supported by increased expression of the proinflammatory factors IL‐1*β*, IL‐6, TNF‐*α*, and MCP1. However, compared with those in the LPS+GelMA group, the expression levels of the proinflammatory‐related genes were clearly downregulated in the LPS+5dECM group, with the relative quantification decreased by 50% compared with the LPS group. These changes were also confirmed by ELISA analysis. In the supernatant, the cytokines IL‐6 and TNF‐*α* secreted by macrophages cocultured with dECM were significantly decreased compared with the LPS‐treated group (Figure 6B).

These in vitro results prompted us to further confirm the anti‐inflammatory function of the dECM bioink by conducting an in vivo experiment. After subcutaneous implantation for 3 days in Sprague Dawley rats, some inflammatory cells had aggregated around the GelMA module, while a few inflammatory cells were observed around the GelMA+5dECM module (Figure 6C). On day 10, a thicker encapsulated fibrous layer had formed around the GelMA module, and a large number of inflammatory cells extensively infiltrated into it, suggesting persistent chronic inflammation. In contrast, the dECM group had significantly less inflammatory infiltration, with a thinner fibrous capsule. Further analysis by IF staining demonstrated a lower proportion of proinflammatory M1 macrophages among the inflammatory cells aggregated around the GelMA+5dECM module than around the pure GelMA module on day 10 (Figure 6D). In addition, the expression of the proinflammatory cytokine IL‐1*β* and the anti‐inflammatory cytokine TGF‐*β* was higher around the pure GelMA module on day 10, as demonstrated by immunohistochemistry (IHC) staining, suggesting a significant inflammatory response and tissue repair processes (Figure 6E). The lower expression of IL‐1*β* and TGF‐*β* in the GelMA+5dECM group confirmed the immunomodulatory function of the dECM. Interestingly, IF and IHC staining in both groups exhibited inconspicuous infiltration of macrophages and inflammatory factors on day 3. This was probably because the graft had not degraded in the early stage of transplantation. As the implantation time increased, the bioinks degraded, and the encapsulated xenogeneic DFCs were released, resulting in local inflammation. These in vitro and in vivo results were consistent and collectively demonstrated that the dECM could be beneficial for generating an anti‐inflammatory microenvironment.

### 2.4 In Vivo Periodontal Regeneration Induced by the 3D Bioprinted Periodontal Module

**Figure 7:**
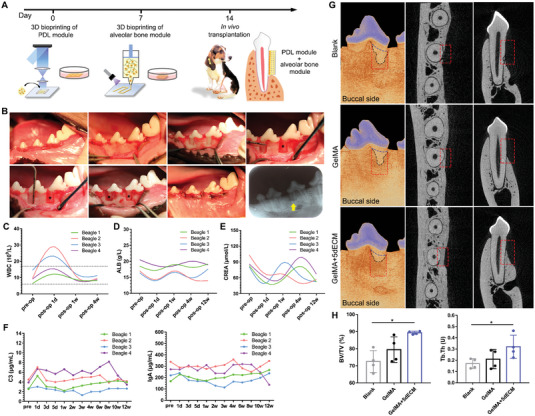
Evaluation of the osteoinduction activities and immune rejection of the bioprinted periodontal module. A) Flowchart of constructing the periodontal module complex. B) Surgical procedure (black asterisk: transplanted periodontal module) and X‐ray image of the periodontal defect (yellow arrow) after surgery on day 0. C) The number of WBCs in routine blood tests before and after surgery (black dotted lines: the normal range). D) The content of ALB and E) CREA in serum biochemical analysis of liver and kidney function before and after surgery. F) ELISA results of complement C3 and immunoglobulin IgA content in serum. G) Micro‐CT scanning of the periodontal defect 3 months after transplantation (red dotted line: defect area; blank dotted line: the boundary of new bone; blue dotted line: natural bone height). H) Quantitative analysis of BV/TV and Tb.Th at 3 months postsurgery based on micro‐CT scanning. (BV/TV: *p = 0.0301; Tb.Th: *p = 0.0280). (Two‐sided comparison, error bars represent standard deviation, *p < 0.05, **p < 0.01 and ***p < 0.001).

According to the aforementioned results, the 3D bioprinted PDL module was cultured for 14 days, and the alveolar bone module was cultured for 7 days in vitro. Then, the two modules were combined and transplanted into the periodontal defect of beagles (**Figure** **7**A). The surgical procedure is shown in Figure 7B, and a well‐defined box‐shaped defect could be seen on the postoperative radiograph. Periodontal scaling and postoperative follow‐up were performed every two weeks (Figure S1, Supporting Information). Swollen gingiva, gingivitis and obvious periodontitis were not observed. The X‐ray images showed that the bone density in the defect area gradually increased while the boundary disappeared. No abnormal alveolar bone resorption was observed. To determine whether the graft would cause systemic immune rejection and damage to liver and kidney function, blood samples were regularly collected for further analysis. Routine blood results demonstrated that the number of white blood cells (WBCs), especially granulocytes, increased 1 day after surgery and returned to normal within 1 week, which was considered to be caused by surgical trauma (Figure 7C; Figure S2A, Supporting Information). The serum biochemical indicators of the liver (Figure 7D; Figure S2B, Supporting Information) and kidney (Figure 7E; Figure S2C, Supporting Information) undulated within a certain range without any abnormalities. The immunological analysis of blood serum also showed no significant abnormalities in the contents of immunoglobulin A, G, or M (IgA, IgG, or IgM, respectively) or humoral immunity markers C3 or C4; they fluctuated within the normal range (Figure 7F; Figure S2D, Supporting Information). In addition, we harvested and stained the major organs of the beagle to assess whether the transplanted cells and materials would cause organ damage and systemic metastasis (Figure S3, Supporting Information). Neither organic lesions in H&E staining nor GFP‐positive cells in IF staining were found in any of the tissues, confirming the safety of our periodontal module. In conclusion, our bioprinted periodontal module did not give rise to apparent immunological rejection or systemic damage in beagles.

**Figure 8:**
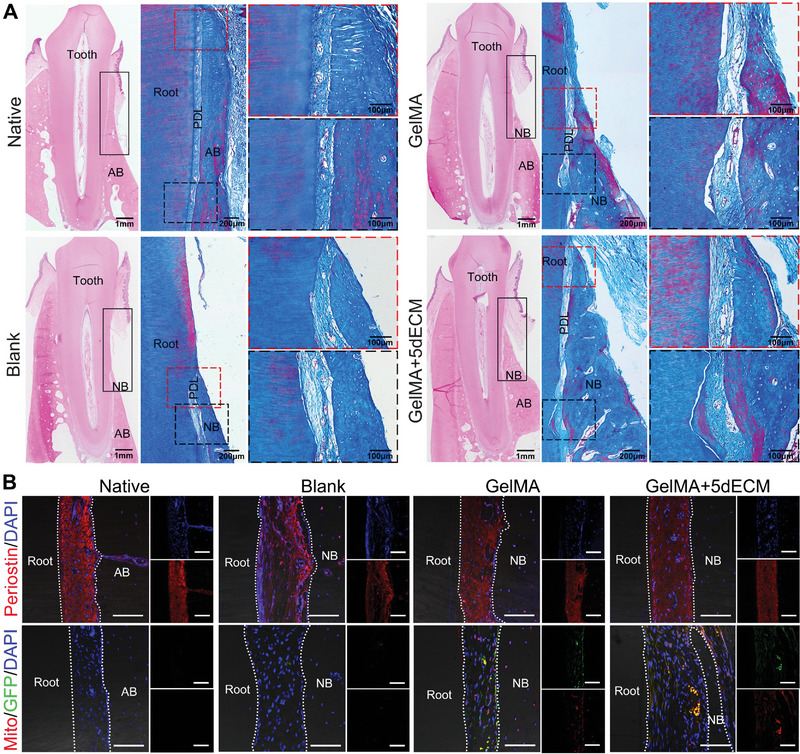
Periodontal regeneration induced by the 3D bioprinted periodontal module. A) H&E and Masson's trichrome staining showed evident new bone and PDL formation in the GelMA+5dECM group (black rectangle: defect area). B) IF staining of the PDL‐specific protein periostin and human cell‐specific protein mitochondria in newly formed tissues (white dotted lines: the bone–ligament and ligament–root interfaces). Scale bars = 100 µm. AB: alveolar bone; NB: new bone; PDL: periodontal ligament.

After 3 months, the micro‐CT results showed poor mineralization and relatively low bone volume/tissue volume (BV/TV) and trabecular thickness (Tb.Th) in the blank samples. However, evident bone regeneration, improved restoration of alveolar bone height and thickness, and substantially increased BV/TV and Tb.Th were observed in the GelMA+5dECM group compared with the other groups (Figure 7G,H). Although the GelMA group also recovered a certain amount of bone mass, the height and thickness were unsatisfactory. Histological staining showed that large amounts of new bone had formed alongside the root surface in the defect receiving the GelMA+5dECM implant, with the presence of soft tissues between the new bone and tooth root (**Figure** **8**A). The regenerated soft tissues observed in both experimental groups all comprised fibroblasts and collagen fibers, which were densely arranged and well organized. Notably, part of the fibers adjacent to the bone surface were inserted into the new bone to generate a hard/soft tissue interface, highly similar to Sharpey's fibers in natural teeth.^[^ ^33^ ^]^ Additionally, a large number of blood vessels were observed in the periodontal clearance in the GelMA and GelMA+5dECM groups. These vessels could provide good nutrient supply for tissue regeneration, but they also had adverse effects on the direction and arrangement of new fibers. Furthermore, IF staining was used to identify the newly formed periodontal ligament tissues (Figure 8B). Periostin, regarded as a specific marker to characterize PDL tissues, was observed in all the experimental groups and the native tooth root, suggesting that the newly formed tissues were indeed PDL fibers.^[^ ^34^ ^]^ The positive expression of human cell‐specific protein mitochondria and GFP in the GelMA and GelMA+5dECM groups indicated that the DFCs encapsulated in the printed module were involved in the formation of PDL and new bone.

**Figure 9:**
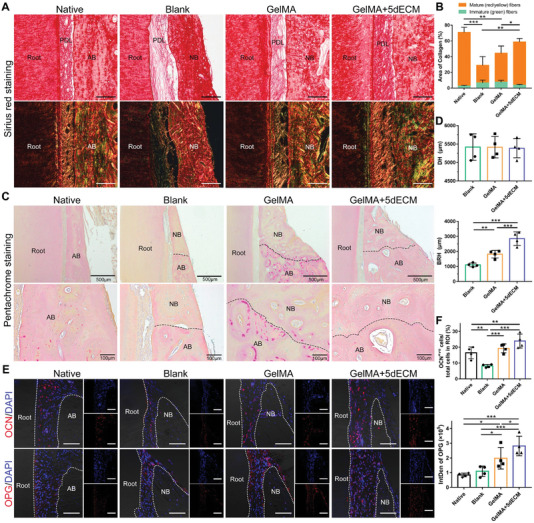
The bioprinted periodontal module constructed by GelMA+5dECM ink led to increased bone regeneration and collagen fiber maturation. A) Sirius red staining under polarized light showing collagen fiber organization in the newly formed PDL. B) Quantification of green pixels (corresponding to immature collagen fibers) and red/yellow pixels (corresponding to mature fibers) in the total area of PDL (*p = 0.0278, **p = 0.0016, **p = 0.0013, ***p = 1.38 × 10^−4^). C) Movat–Russell modified pentachrome staining showing the interface between new bone and natural bone (black dotted lines). D) Histometric analysis of defect height (DH) and bone regeneration height (BRH) in tissue slices from the three groups (**p = 0.0095, ***p = 2.1 × 10^−5^, ***p = 9.91 × 10^−4^). E) IF staining of the osteogenic markers OCN and OPG at the alveolar ridge crest (white dotted lines: the bone–ligament and ligament–root interfaces). F) Quantification of the percentage of OCN^+ve^ cells/total cells and the integrated density of OPG in an ROI (OCN: **p = 0.0035, **p = 0.0070, ***p = 3.93 × 10^−4^, ***p = 1.7 × 10^−5^; OPG: *p = 0.0103, *p = 0.0358, *p = 0.0453, ***p = 1.98 × 10^−4^, ***p = 6.14 × 10^−4^). Scale bars = 100 µm. * *p* < 0.05, ** *p* < 0.01 and *** *p* < 0.001. AB: alveolar bone; NB: new bone; PDL: periodontal ligament. (Two‐sided comparison, error bars represent standard deviation, *p < 0.05, **p < 0.01 and ***p < 0.001).

To further evaluate collagen maturation in nascent PDL, Sirius red‐stained slices were observed under polarized light to distinguish mature and immature fibers (**Figure** **9**A). The collagen matrix in the GelMA+5dECM group appeared to be more mature, and its area was larger, followed by that of the GelMA group (Figure 9B). The area of collagen in the blank group was the lowest and mainly comprised immature collagen. In addition, the newly formed PDL fibers exhibited good orientation and uniform arrangement in the GelMA+5dECM group, similar to the native tooth (Figure 9A). Movat–Russell modified pentachrome staining showed an evident interface between new bone and natural bone in the blank and GelMA groups (Figure 9C). In the GelMA group, some immature bone units were noticed in the new bone adjacent to the interface, but none were observed in the blank group. In contrast, the interface in the GelMA+5dECM group was not clear, and larger Haversian systems could be observed in the new bone area, verifying higher bone mineralization.^[^ ^35^ ^]^ Furthermore, the bone regeneration height (BRH) at 3 months postsurgery was quantified using pentachrome staining images. Based on the statistically nonsignificant defect height (DH), defects receiving either implant exhibited robust bone regeneration; however, the GelMA+5dECM module led to higher BRH values than the GelMA module (Figure 9D). Moreover, cells positive for the late osteogenic marker OCN were present at certain densities in regenerated tissues arising from defects that received either GelMA or GelMA+5dECM modules but were almost absent in the blank group (Figure 9E,F). The expression of OPG protein, which inhibited the formation and activity of osteoclasts, was significantly higher in the GelMA+5dECM group than in the GelMA and blank groups, suggesting higher osteogenic potential.^[^ ^36^ ^]^ Collectively, the bioprinted periodontal module constructed by GelMA+5dECM bioink promoted the regeneration of functional periodontal tissues, including higher alveolar bone recovery, more mature PDL fibers, and a more sophisticated fusion of the interface.

## 3 Discussion

We developed a biomimetic and functional 3D bioprinted module that mimics the composition and structure of a natural periodontal composite to promote fibrogenesis and osteogenesis. The unique feature of the functional module was the biochemical component and odontogenic microenvironment provided by dental follicle‐derived dECM. It succeeds in improving the mechanical property and bioactivity of the hybrid bioink, which contributes to cell survival, biological behaviors, and tissue remodeling. Well‐aligned periodontal fibers, highly mineralized alveolar bone and satisfactory bone–ligament interface integration were achieved by the bioprinted module after 3 months of implantation in vivo, and the absence of immune rejection and systemic lesions ensured safety.

3D bioprinting technology or additive manufacturing strategies have made significant progress in tissue engineering and regenerative medicine. Cell‐laden hydrogels, serving as bioinks, provide a microenvironment resembling the ECM for cell bioactivity.^[^ ^37^ ^]^ To date, several types of natural and synthetic materials, such as collagen, gelatin, alginate, silk fibroin and polyethylene glycol, have been used as bioinks.^[^ ^37^, ^38^, ^39^, ^40^ ^]^ Among them, GelMA has been considered for various tissue constructs due to its suitable viscosity, strong formability and good biocompatibility and biodegradability.^[^ ^41^, ^42^ ^]^ In 2016, Yelick's research group confirmed the feasibility of GelMA hydrogels in the field of tooth regeneration.^[^ ^43^ ^]^ Nevertheless, no single‐component bioink can precisely represent the intricate microenvironment of native ECM. Thus, dECM‐derived bioinks, which facilitate the retention of the natural ECM complexity that promotes cell activity and tissue regeneration, have been introduced.^[^ ^20^, ^44^, ^45^ ^]^ However, the low viscosity of the dECM bioink leads to weak formability and makes manufacturing complex structures difficult.^[^ ^30^, ^46^ ^]^ To address this issue, in this study, dECM was incorporated into a GelMA hydrogel to form a hybrid bioink. The mechanical and biofunctional analysis of the GelMA/dECM bioink exhibited both advantages of good printability and excellent bioactivity (Figures 2 and 3), which exactly satisfied our demands, suggesting an appropriate candidate for periodontal regeneration based on 3D bioprinting.

Because of the complexity of the periodontal structure, choosing a 3D bioprinting method is difficult. Existing 3D bioprinters mainly include inkjet bioprinters and extrusion bioprinters based on DIW and photocurable bioprinters based on DLP.^[^ ^47^ ^]^ The printing accuracy of DIW printers depends on the size of the nozzle, and its advantage lies in the rapid construction of large‐scale spatial structures.^[^ ^48^ ^]^ Due to the low printing resolution, extrusion bioprinters have mainly been used for larger organs with uniform structure and composition, such as bone, kidney, heart, and muscle.^[^ ^11^, ^15^, ^24^ ^]^ DLP‐based bioprinters have the advantages of higher‐resolution preparation of hydrogels with detailed architecture and less cell damage, and they have been used to successfully reconstruct sophisticated structures, such as nerve conduits, hepatic lobules, and capillary networks.^[^ ^16^, ^49^, ^50^ ^]^ Unfortunately, our team's existing DLP bioprinter cannot move on the *z*‐axis. It can only print a single layer in *x*–*y* plane, failing in constructing the spatial grid structure of the alveolar bone module. Although DIW printers are able to move arbitrarily in 3D space, it is difficult to achieve an accuracy below 100 µm with existing technology. Considering our existing equipment and the different demands of spatial structure, we finally chose different bioprinting methods to construct two modules. Simulating the dimension and orientation of natural periodontal fibers, hydrogel pillars with a diameter of 150 µm were fabricated by a DLP bioprinter (Figure 4). In our initial design, the single columnar structure was prone to collapse and deformation during in vitro culture and could not maintain stability. Good results were achieved by adding a square grid around the pillars to ensure that the pillars were upright and a gap was maintained to facilitate nutrient exchange. The width of the grid was only 40 µm, making it difficult for DFCs to grow and stretch inside such that directional alignment along the hydrogel pillar of DFCs was achieved. The decussate grid structure of the alveolar bone module was formed by an extrusion bioprinter (Figure 5); the structure can not only obtain a certain volume of space but also ensure the nutrient supply in the middle. The feasibility of this structure has been confirmed by several studies and is regarded as a classic design in bone tissue engineering.^[^ ^51^, ^52^ ^]^

Studies have demonstrated the tissue‐specific induction potential of the dECM, summarized the natural microenvironment of tissues and provided clues to drive cell proliferation and differentiation.^[^ ^21^, ^22^, ^23^ ^]^ The dECM can retain tissue‐specific growth factors, cytokines, adhesion proteins, and signaling molecules, which participate in the regulation of cellular behavior, such as migration, proliferation, differentiation, and signaling.^[^ ^53^ ^]^ In our research, the porcine dental follicle was selected due to its anatomical and biochemical similarity to the human dental follicle. What's more, the massive size of the porcine dental follicle also provides an inexpensive and readily available source of tissues for tooth regeneration.^[^ ^54^, ^55^ ^]^ Additionally, the plentiful provision of animal tissue, especially porcine tissue, supplies a potential solution to the desperate shortage of human tissue in terms of material affordability.^[^ ^56^ ^]^ As predicted, the porcine dental follicle‐derived dECM supported tissue‐specific function and promoted the proliferation, migration, and specific odontogenic differentiation of DFCs (Figure 3). In this study, the addition of dECM into the GelMA bioink significantly improved the cell viability of DFCs in the printed alveolar bone modules (Figure 5B). Considering the resolution limitation of the extrusion printer, the practical diameter of the printed strand was 410.61 ± 26.73 µm, while the diffusion limit of nutrients and oxygen in hydrogels was 100–200 µm.^[^ ^57^ ^]^ This led to direct interference with the survival and proliferation of DFCs in the GelMA hydrogel. However, the addition of dECM might provide a microenvironment favorable to survival and capable of mitigating this negative effect, and the results were consistent with previous research findings.^[^ ^24^, ^50^ ^]^ This conjecture was also supported by the result showing that the cell viability was not significantly different between the two groups with the PDL module (Figure 4C), where adequate nutrient exchange was obtained due to the fine structure.

The presence of xenogeneic/allogeneic DNA residues and exposure of cell membrane antigenic epitopes in the dECM might trigger an adverse immune response, while multiple macromolecules in the dECM also play key roles in tissue remodeling and regeneration.^[^ ^58^, ^59^ ^]^ As a biophysical and biochemical microenvironment, the dECM has a great influence on the behavior of macrophages. Interestingly, several studies have confirmed that the dECMs derived from various tissues exhibited different polarization effects on macrophage phenotypes.^[^ ^60^ ^]^ Dziki et al. determined the effects of dECMs derived from eight different source tissues on macrophages and found that the dECMs obtained from the urinary bladder, esophagus, small intestinal submucosa, brain, and colon induced an M2‐like macrophage phenotype, while dermal dECM resulted in an M1‐like phenotype, but dECM from skeletal muscle and liver had no significant effect.^[^ ^61^ ^]^ Other studies noted that both decellularized porcine periosteum and the brain promoted macrophage chemotaxis and M2 polarization.^[^ ^62^, ^63^ ^]^ Another study, however, reported that dECM from the urinary bladder elicited the M2‐like macrophage response attributed to its abundant hyaluronic acid component, while the brain dECM, lacking hyaluronic acid, induced macrophages to an M1‐like phenotype.^[^ ^64^ ^]^ In the present study, porcine dental follicle‐derived dECM suppressed M1 macrophage activation to generate an anti‐inflammatory microenvironment (Figure 6) and did not give rise to apparent immunological rejection in beagles (Figure S2). All of the aforementioned results have demonstrated the immune regulation of macrophage polarization by the dECM hydrogel; unfortunately, the specific mechanism and functional components remain unclear. Sicari et al. proposed that the degradation products of the dECM bioscaffold, in the form of bioactive oligopeptides, promoted the polarization of constructive macrophages.^[^ ^65^ ^]^ However, extensive additional work is necessary to identify specific cryptic peptides responsible for the modulation of macrophage phenotype.^[^ ^65^ ^]^

The most exciting result of the present study was that the bioprinted periodontal module incorporating dECM significantly promoted the regeneration of hybrid periodontal tissues, especially the anchoring structures of the bone–ligament interface, well‐aligned periodontal fibers and highly mineralized alveolar bone (Figures 8 and 9). The complex structure of the periodontium, wherein the PDL is inserted in a specific orientation into the cementum and alveolar bone, makes the regeneration of periodontal tissue extremely difficult.^[^ ^5^, ^6^ ^]^ In 2012, Park et al. manufactured a biomimetic fiber‐guiding scaffold using a computer‐aided 3D printer to guide functional periodontal reconstruction, which was the first time that 3D printing technology had been applied to periodontium regeneration.^[^ ^66^ ^]^ However, limited by the poor printing precision and slow degradation rate of polycaprolactone (PCL), the osteogenic effect of the biomimetic scaffold was unsatisfactory, and only PDL‐like fibrous tissues were formed. In 2016, another PCL scaffold was designed by Pilipchuk et al. to integrate a 3D‐printed bone region with a micropatterned PCL thin film consisting of grooved pillars.^[^ ^67^ ^]^ The results showed enhanced oriented collagen fiber thickness using micropatterned PCL films but poor bone regeneration surrounding the undegraded PCL bone region. After several attempts, 3D printing technology has thus far failed to achieve great success in the field of periodontal tissue engineering, which is mainly due to the limitations of scaffold materials and printing conditions.^[^ ^68^, ^69^ ^]^ Balancing the degradation rate of ink with the regeneration rate of periodontal tissue and developing a more sophisticated 3D printer to achieve the anatomical accuracy of PDL are the primary obstacles. Fortunately, with the rapid advance of printing technologies and materials science, 3D printing has entered a new era with 3D bioprinting technology. Here, we integrated dECM bioink with 3D bioprinting technology for periodontal tissue remodeling for the first time and succeeded in recovering a more mature PDL–alveolar bone complex. Most importantly, the regenerated fibers near the bone surface were oriented perpendicularly and inserted into the newly formed alveolar bone. These fibers were very similar to Sharpey's fibers in native PDL, which are known to insert into the tooth root at a certain angle, and this structure would reinforce the stability of the tooth root in the jaws and provide the necessary mechanical loading.^[^ ^33^ ^]^ The successful reproduction of this soft‐hard tissue interface in the present study further verified the effectiveness and feasibility of our bioprinted periodontal module constructed by GelMA/dECM bioink. In addition, the GelMA/dECM group still exhibited active osteogenic potential after 3 months of implantation, with evidence of positive expression of the osteogenesis markers OCN and OPG at the alveolar ridge crest (Figure 9), enabling continued alveolar bone height reestablishment with the extension of transplantation time.

In summary, our functional module mimicked both the biochemical microenvironment and anatomical features of natural periodontal tissue by modifying the bioink composition and optimizing the structure design. We hypothesized that the mechanism underlying its excellent periodontal regeneration potential was twofold. On the one hand, the physical properties such as the spatial structure and microscopic morphology of the designed module might be adapted to the mechanical forces in the encapsulated DFCs.^[^ ^70^ ^]^ Then the mechanical signals were converted into chemical signals to further promote the odontogenic differentiation of stem cells. On the other hand, the residual tissue‐specific biochemical components in dECM, including growth factors, cytokines, proteoglycan, and structural and adhesion proteins, provided a microenvironment similar to native tissues and participated in the regulation of cellular behavior.^[^ ^53^ ^]^ Given the remarkable osteogenic ability of the alveolar bone module in this study, it may have a great application prospect in the repair and regeneration of craniomaxillofacial bone defects. Our functional periodontal module may also serve as a drug detection chip or bioreactor to analyze the effects of different drugs, bacteria or other biomolecules on periodontal regeneration or destruction in vitro. In addition, the successful fusion of PDL–alveolar bone interface in the regenerated periodontal tissue also lights up new ideas for the reconstruction of biphasic structures such as bone–cartilage and bone–tendon interface.

While we observed an outstanding regeneration effect of the bioprinted GelMA/dECM module in periodontal defect recovery, some challenges remain to be addressed in the future. For example, constructing a periodontal module using two different types of printing methods was inconvenient, and this could become a serious obstacle to future clinical applications. A recent study reported a DLP‐based bioprinting system able to produce sophisticated hydrogel constructs with both horizontal and vertical gradients in both 2D and 3D at reasonably high resolutions.^[^ ^49^ ^]^ The launch and commercialization of this new bioprinter in the future could solve our problem of using two printing methods. Another limitation is that the newly formed cementum accrued mainly on the dentin surface at the bottom of the defect, and only a few new cementum were present near the alveolar ridge crest. The existence of cementum is crucial for the orientation of PDL, while intact cementum regeneration has not yet been achieved.^[^ ^71^ ^]^ Further studies are needed to utilize the cementum module in the existing periodontal module to construct the sandwich periodontal complex with integrated and functional periodontal tissue.

## 4 Conclusion 

This study has developed a biomimetic and functional periodontal module using the GelMA/dECM bioink by employing a 3D bioprinting approach. The module could simulate natural anatomical complexity and biochemical microenvironment, and exhibit strong biological induction activity. It suppressed the M1 macrophage activation and induced fiber orientation and osteogenesis, thus leading to an integrated periodontal regeneration in Beagle models. Collectively, our bioprinted periodontal module using the dental follicle‐specific dECM bioink provides a promising clinical alternative for periodontal regeneration.

## 5 Experimental Section

### Preparation and Characterization of the Dental Follicle‐Derived dECM

Porcine dental follicles were harvested from the alveolar bone of pigs at a local slaughterhouse (Chengdu, China). The tissues were cut into small pieces and continuously rinsed in sterile phosphate‐buffered saline (PBS) for 24 h. Then, the samples were treated with sterile 1% Triton X100 solution at 4 °C for 12 or 24 h and washed with sterile PBS for another 2 h. After soaking in 100 U mL^−1^ DNase I at 37 °C for 12 or 24 h, the samples became transparent. Finally, the samples were washed with sterile PBS for 2 days to remove the enzyme and fragments in the decellularized matrix and then stored at 4 °C for further use.

Both native and decellularized dental follicle tissues (Triton X100 and DNase I treated for 12 and 24 h) were fixed with 4% paraformaldehyde, dehydrated in a series of ethanol solutions, cleared with xylene and embedded in paraffin. Then, cell component residues were observed by hematoxylin and eosin (H&E) staining. Collagen distributions were observed by Masson's trichrome staining (Baso Diagnostic Inc., China). The sections were then incubated with 100 ng mL^−1^ DAPI for 10 min to stain the nuclei. IHC staining was also used to examine ECM components. The primary antibodies were anti‐COL1 and antifibronectin (1:200 dilution, Abcam, UK), and the secondary antibodies were visualized using a DAB kit (Gene Tech, China). The surface morphology of the three samples was analyzed with SEM (Inspect F, FEI, USA). The amount of double‐stranded DNA (dsDNA) was quantified using a DNA extraction kit (TIANGEN, China). The dsDNA concentration was determined using a Nanodrop instrument (Thermo Fisher Scientific, USA). The total sGAG and total collagen contents were estimated by utilizing the Blyscan sGAG assay kit (Biocolor, UK) and hydroxyproline assay kit (Solarbio, China), respectively. All procedures were performed according to the manufacturer's protocols.

### Preparation of the GelMA/dECM Bioink

The dECM of dental follicle tissue was generated and processed as previously described.^[^ ^13^ ^]^ Lyophilized dECM was ground into powder, digested in 0.5 m acetic acid and pepsin solution (15 mg pepsin per 100 mg dECM) for 4 days. After complete digestion, a dECM solution was obtained by centrifuging at 4000 rpm for 10 min to remove the particles. Then, 10 m cold NaOH solution was added to the dECM solution to adjust the pH to ≈7.4. The pH‐adjusted dECM solution was stored at 4 °C for further use. GelMA solutions were created by dissolving lyophilized GelMA and lithium phenyl‐2,4,6‐trimethylbenzoylphosphinate (LAP, Engineering for Life, EFL, China) in *α*‐MEM. Finally, the dECM solution was mixed with the GelMA solution to form GelMA/dECM bioinks with concentrations of 10% (w/v) GelMA+2.5 mg mL^−1^ dECM, 10% (w/v) GelMA+5 mg mL^−1^ dECM and 10% (w/v) GelMA+10 mg mL^−1^ dECM. A 10% (w/v) GelMA solution was used as a control.

### Mechanical Properties of the GelMA/dECM Bioink

To obtain information regarding the rheological behavior of the GelMA/dECM bioink, viscoelasticity analysis was performed using an HAAKE Viscotester iQ Air (Thermo Scientific). The C35 1°/Ti cone rotator was used to perform the experiments with a truncation gap distance of 1 mm. Shear rate sweeps were performed to determine the shear viscosity of the GelMA/dECM bioink by changing the applied shear rate from 0.1 to 100 s^−1^ at 25 °C. Frequency sweeps were conducted to characterize the dynamic modulus of the bioink. The applied strain constant was determined to be 0.01 over the frequency range of 0.1–10 Hz at 25 °C. The swelling and degradation properties of GelMA hydrogels with and without dECM were also measured. The hydrogel samples were manufactured in a cylindrical shape with the same volume (400 µL) and exposed to 405 nm ultraviolet (UV) light for 30 s. The power of the LED light source was 5 W in this study. For the swelling test, the samples were incubated in PBS at 37 °C for 24 h. After removal from PBS, the wet weight of the samples (*W*_s_) was determined. The samples were then lyophilized for 8 h, and the dry weight (*W*_d_) was determined. The swelling ratios of the hydrogels were obtained by the equation *Q*_s_ = (*W*_s_ − *W*_d_)/*W*_d_. For the degradation test, the samples were placed in a 24‐well plate with 0.5 mg mL^−1^ collagenase I (HyClone, USA) at 37 °C, and samples were taken at each preselected time point (0, 6, 12, 18, and 24 h, *n* = 3). Then, we lyophilized each sample for 8 h to obtain the remaining weight (*W*_r_). Considering the weight at 0 h as *W*_0_, the biodegradation ratios (*Q*_d_) of hydrogels were obtained by the equation *Q*_d_ = *W*_r_/*W*_0_ × 100%.

### Printability Analysis of the GelMA/dECM Bioink

Four types of hydrogel samples were tested using a 3D‐Bioplotter System (EnvisionTEC, Germany) fitted with a 30CC barrel in a clean bench. During the extrusion process, a tapered plastic tip was utilized. The inner diameter of each needle was 260 µm. The temperature of the barrel that loaded the bioink was 27 °C, and the room temperature was 23 °C. The initial extrusion pressure was 10 kPa, and the pressure was gradually increased (10 kPa each time) until the bioink could be extruded. The minimum pressure required for extruding the bioink from the barrel was recorded. Moreover, we observed whether the bioink could be continuously and uniformly extruded.

The mass flow rate of the bioink was measured first. The four groups of bioinks were deposited on weighing paper under their stable bioprinting pressure (5 s), and the paper containing bioinks was weighed by a precise balance immediately after deposition. The effect of bioprinting speed on the diameter of printed filaments was evaluated. The bioinks were loaded into the barrel and printed at their respective stable extrusion pressure and fixed flow rate. The filaments created at different printing speeds (6, 8, 10, and 12 mm s^−1^) were printed into dishes and exposed to 405 nm UV light for 30 s. The filaments were observed under an optical microscope (Olympus, Japan) to measure the diameter (*D*_real_). The theoretical diameter (*D*_theo_) could be calculated according to the mass flow rate, and the differences were obtained as *D*_real_/*D*_theo_.

The assessment of shape fidelity was performed as previously described.^[^ ^30^ ^]^ Four types of hydrogel samples were used to print a two‐layered grid construct with a layer distance of 200 µm. Bioinks should be printed at a similar flow rate. The bioprinting speed was set to 6, 8, 10, and 12 mm s^−1^ to construct a two‐layer scaffold. For the internal structure, the distance between two adjacent filaments was 1.2 mm. After being exposed to 405 nm UV light for 30 s, the structure of the prints was observed under an optical microscope (Olympus, Japan). The pore size of the scaffold was measured to characterize the printability of the bioink. Three images were randomly collected from each scaffold, and three pores were randomly selected from each image and analyzed by ImageJ software. The shape fidelity (SF) of hydrogels was obtained by the equation SF = *A*_RE_/*A*_TE_ × 100%, where *A*_RE_ is the area of real empty space and *A*_TE_ is the area of theoretical empty space.

### Cell Culture

Impacted third molars obtained from 16‐ to 20‐year‐old healthy young patients whose teeth were extracted for clinical reasons were collected for cell isolation. All experiments were conducted in accordance with the ethical protocol approved by the Committee of Ethics of Sichuan University, and written informed consent was obtained from all guardians on behalf of the teenagers enrolled in this study. Dental follicles of impacted third molars were dissected and rinsed with sterile PBS. The tissues were cut into 1 × 1 mm blocks and incubated in *α*‐MEM supplemented with 10% fetal bovine serum (FBS, HyClone, USA) in a humidified 5% CO_2_ atmosphere at 37 °C. The cell culture medium was replaced every 2 days. DFCs were used at passages 3–5 in all experiments.

### Evaluation of the Biofunctionality of the GelMA/dECM Bioink

DFCs were mixed with each of the four bioink solutions, producing a final concentration of 2.5 × 10^6^ cells mL^−1^. The bioinks were then manufactured in a cylindrical shape with the same volume (100 µL) and exposed to 405 nm UV light for 30 s. Cell viability was evaluated based on fluorescent observation using a Live/Dead Viability/Cytotoxicity kit (KeyGEN, China) after culturing for 1, 3, and 7 days in vitro. Six randomized images from each group were captured and imported into ImageJ software for cell counting. The Cell Counting Kit‐8 (CCK‐8; Dojindo, Japan) was used to quantitatively evaluate the proliferation of DFCs encapsulated in bioink after 1, 3, 5, and 7 days of culture, and the optical density (OD) values at 450 nm were determined using a spectrophotometer (Thermo Fisher Scientific, USA). The expression of the osteogenic genes Runx2, ALP, OPN, and COL‐1 and the odontogenic genes DSPP, periostin, and laminin‐*β*1 was analyzed using real‐time PCR after 7 days of culture. The primer sequences are listed in Table S2 (Supporting Information). Relative expression levels were calculated using the 2^−ΔΔCT^ method and normalized to the reference h‐GAPDH gene.

Cell migration was determined using a Chemotaxicell chamber (8 µm pore size, Costar, USA). After serum starvation for 24 h, the DFC suspension (100 µL; 1 × 10^6^ mL^−1^) was added to the upper chamber. Then, 500 µL of various bioink diluents (serum‐free culture medium containing 0.25, 0.5, 1 mg mL^−1^ dECM or 1% GelMA) were separately added to the lower chamber and incubated at 37 °C for 12 h. Cells that migrated to the lower surface of the membrane were fixed with 4% paraformaldehyde and stained with 1% crystal violet.

### Construction and Biological Function Analysis of the PDL Module

We designed a PDL module 400 µm thick consisting of arrays of hydrogel pillars and square grids. The hydrogel pillar mimicked the oriented periodontal fibers, and the square grid provided mechanical support to prevent collapse. The internal diameter of each unit was 250 µm, including a hydrogel pillar 150 µm wide and a 50 µm gap around it. The square grid was 40 µm in width. The DLP 3D printing system was provided by the State Key Laboratory of Biotherapy and Cancer Center, Sichuan University. We sandwiched two narrow silicon membranes between two holders to obtain a gap, which controlled the thickness of the module. With the digital 3D images set up in the computer, GelMA/dECM bioink with a DFC density of 2 × 10^6^ mL^−1^ was subsequently added to the designed gap. After exposure for 10 s, the PDL module was obtained. To better display the printed structure, fluorescein isothiocyanate (FITC)‐labeled GelMA was used and observed under a laser scanning confocal microscope (Olympus, Japan).

To detect viable cells in the PDL module after 3, 7, and 14 days of culture, a Live/Dead Viability/Cytotoxicity kit (KeyGEN, China) was used. The samples were observed and reconstructed in three dimensions under a laser scanning confocal microscope (Olympus, Japan). For the quantification of cell viability, four randomized images from each group were captured and imported to ImageJ software for cell counting. On day 7, the PDL module was fixed with 4% paraformaldehyde, and IF staining was performed to determine whether dECM promotes fibroblast differentiation. The samples were incubated with primary antibodies (antifibronectin and antilaminin, 1:200 dilution, Abcam, UK) at 4 °C overnight and secondary antibodies (Alexa Fluor 488‐conjugated goat antimouse and Alexa Fluor 555‐conjugated goat antirabbit, 1:200 dilution, Invitrogen, USA) for 1 h at room temperature. Next, the samples were incubated with 100 ng mL^−1^ DAPI for 5 min to stain the nuclei. All samples were examined under a laser scanning confocal microscope (Olympus, Japan).

### Construction and Osteogenic Property Analysis of the Alveolar Bone Module

Using a DIW 3D‐Bioplotter System (EnvisionTEC, Germany), a cube structure (10 × 10 × 2.5 mm) with an inner strand distance of 0.9 mm and inner strand angles of 0° and 90° was built to mimic alveolar bone. DFCs were mixed with two bioinks at a density of 5 × 10^6^ cells mL^−1^. The needle offset along the *z*‐direction was 210 µm, which was 80% of an ideal strand diameter (260 µm) for each printed layer. The printing pressure was set as 90 kPa for the GelMA bioink and 150 kPa for the GelMA+5dECM bioink. The printing speed was 12 mm s^−1^, and the printed samples were finally exposed to 405 nm UV light for 30 s.

After culturing for 1, 3, and 7 days in vitro, cell viability was evaluated using a Live/Dead Viability/Cytotoxicity kit (KeyGEN, China). Seven fields were randomly selected from each group for quantitative analysis. To detect osteogenic differentiation in the alveolar bone module, IF staining was performed at 7 and 14 days. The primary antibodies included anti‐ALP (1:100 dilution, Abcam, UK), anti‐Runx2 (1:100 dilution, Abcam, UK) and anti‐OCN (1:200 dilution, Zenbio, China). All samples were examined under a laser scanning confocal microscope (Olympus, Japan).

### Anti‐inflammatory Effect of dECM In Vitro and In Vivo

A total of 1 × 10^5^ RAW264.7 cells were seeded into each well of a 12‐well plate. RPMI‐1640 containing 10% FBS and 100 U mL^−1^ penicillin/streptomycin was used as the culture medium. Upon reaching 80% confluence, macrophages were treated with different culture media supplemented with 1 µg mL^−1^ LPS, 1 µg mL^−1^ LPS+1% GelMA, or 1 µg mL^−1^ LPS+0.5 mg mL^−1^ dECM for 3 h. RPMI‐1640 containing 10% FBS served as the negative control. Then, the cells were subjected to serum starvation for another 6 h. The supernatant was collected, and the concentrations of the inflammatory factors IL‐6 and TNF‐*α* were detected by an ELISA kit (Deco, China). The gene expression of the proinflammatory factors IL‐1*β*, IL‐6, TNF‐*α* and MCP1 was analyzed using real‐time PCR. The primer sequences are listed in Table S2 (Supporting Information). Relative expression levels were calculated using the 2^−ΔΔCT^ method and normalized to the reference m‐GAPDH gene.

All animal experiments described in this study were conducted in accordance with protocols approved by the Ethics Committee of Sichuan University (ID: WCHSIRB‐D‐2021‐622). Sprague Dawley rats were obtained from DS Experimental Animals Co. Ltd. The alveolar bone module printed using GelMA and GelMA+5dECM bioink was transplanted into the dorsum of Sprague Dawley rats (8‐week‐old males, *n* = 8) under general anesthesia to further verify the anti‐inflammatory effect of dECM in vivo. Each rat was implanted with two samples; one sample was harvested at 3 days, and the other was harvested at 10 days (*n* = 4). The samples were fixed with 4% paraformaldehyde overnight, dehydrated in a series of ethanol solutions and embedded in paraffin. Paraffin sections were prepared and subjected to H&E, IF, and IHC staining. CCR7 and Alexa Fluor 555‐conjugated goat antirabbit antibodies (1:200 dilution, Abcam, UK) were used to identify M1 macrophages by IF staining. IHC staining was performed to detect the infiltration of inflammatory factors, including IL‐1*β* (1:100 dilution, Abcam, UK) and TGF‐*β* (1:200 dilution, Abcam, UK), into tissues. PBS was used in place of the primary antibodies as the negative control.

### In Vivo Periodontal Regeneration

To directly track the distribution and differentiation of DFCs in vivo, lentiviruses encoding eGFP (Hanbio, China) were used to label the DFCs. The printed PDL module encapsulating DFCs‐GFP^+^ was cultured for 14 days, and the alveolar bone module with DFCs‐GFP^+^ was cultured for 7 days. Then, the two modules were integrated, and orthotopic transplantation was performed. The bilateral third and fourth premolars of the beagle (1‐year‐old males, *n* = 4) were selected and randomly divided into four separate groups (*n* = 4): 1) native, 2) blank, 3) GelMA, and 4) GelMA+5dECM. After anesthetization, the gums were incised from the neck of the premolars, and a mucoperiosteal flap was created to expose the alveolar bone of the mandible. The surgical defects, which were ≈6 mm in height, 4 mm in width, and 3 mm in depth, were generated on the mesial buccal side. Scraping the root surface, the composite module was then implanted into the defect, with the PDL module facing the tooth root. The gingiva was carefully sutured after surgery, and the animals received antibiotics for 3 days after surgery. Periodontal scaling was performed every two weeks.

### Micro‐CT and Histological Analysis

The animals were sacrificed after 3 months, and the mandibles were harvested and fixed with 4% paraformaldehyde. Micro‐CT analysis was conducted to evaluate the regeneration of alveolar bone. A 6 × 4 × 3 mm cube was selected as the region of interest (ROI) for quantification of BV/TV and Tb.Th. After decalcification in 17% EDTA, dehydration, and embedding in paraffin, specimens were sliced at a thickness of 5 µm. H&E, Masson's trichrome, IF, Sirius red (Saint Bio, China), and Movat–Russell modified pentachrome (Solarbio, China) staining were performed according to the manufacturer's recommended protocols. The coronal section of the lowest point of new bone was selected for all groups. The antibodies used for IF included periostin (1:300 dilution, Santa Cruz, USA), mitochondria (1:400 dilution, Abcam, UK), OCN (1:300 dilution, Zenbio, China), and OPG (1:200 dilution, Abcam, UK).

To evaluate collagen organization in newborn PDL, Sirius red‐stained slides were viewed under polarized light using an Olympus digital image system. With the color analysis tool in Adobe Photoshop, the orange/red pixels, corresponding to mature collagen fibers, were selected using a uniform color range and counted. The same was done for green pixels, which corresponded to immature collagen fibers. Then, the total pixels within the PDL were determined. Mature/immature collagen fibers were expressed as a percentage of their respective pixels/total pixels.

Histometric analysis according to pentachrome staining was conducted to assess bone regeneration. The DH was the distance from the alveolar ridge crest to the bottom of the defect, and BRH was the distance from the top of the new bone to the bottom of the defect. To evaluate OCN expression, the lasso tool in Photoshop was used to select an ROI, which was the alveolar ridge crest and surrounding PDL. Within this ROI, the number of OCN‐positive cells and the total number of the other cells (identified by DAPI) were then counted, and the ratio of OCN^+ve^ to total cells was then calculated. OPG expression was analyzed by integrated density using ImageJ.

### Biocompatibility and Safety Assessment

To investigate the biocompatibility of dECM, routine blood examination and liver and kidney function tests were performed before and after surgery. Routine blood tests included quantifying WBCs, granulocytes, lymphocytes, and monocytes. Liver function tests included measuring the levels of alanine aminotransferase (ALT), aspartate aminotransferase (AST), albumin (ALB), total protein (TP) and total bilirubin (TBIL). Serum creatinine (CREA), urea, and uric acid (UA) levels were considered indicators of kidney function. We also performed an immunological examination to understand the immune response of graft materials in beagles. The immunological indexes included C3, C4, IgA, IgG, and IgM. All blood samples were sent to LL Biotechnology Co., Ltd., for testing, and the corresponding report was issued.

To determine whether the graft material caused organic lesions, the heart, liver, spleen, kidney, and pancreas of the beagles were harvested and fixed with 4% paraformaldehyde for 48 h. All samples were then embedded and sectioned into sections 5 µm thick for histological analyses. GFP fluorescence labeling was used for IF detection to estimate whether the transplanted DFCs had systemic metastasis.

### Statistical Analysis

Each experiment was repeated independently at least three times with similar results, and the results could be reproduced according to this method. All data are presented as the means ± SDs. Statistical significance was analyzed using SPSS 20.0 software (SPSS, USA). Student's paired t test and one‐way ANOVA were used to determine the level of significance. A value of *p* < 0.05 was considered statistically significant.

## Conflict of Interest

The authors declare no conflict of interest.

## Author Contributions

X.T.Y. and Y.M. contributed equally to this work. X.T.Y. contributed to the design, execution, and analysis of all experiments and wrote the manuscript. Y.M. performed the design and printing of the PDL module and modified the manuscript. X.T.W. and S.M.Y. contributed to animal studies and tissue processing. F.J.H. performed the micro‐CT analysis. G.Z.Y. and J.Y.Z. contributed to the in vitro cell experiment. B.Y. provided research guidance and revised the manuscript. W.D.T. reviewed the manuscript.

## Supporting information

Supporting Information

## Data Availability

The data that support the findings of this study are available in the main text and the supplementary materials or from the corresponding authors upon reasonable request.
