# Supplementary material for: A 3D‐Bioprinted Functional Module Based on Decellularized Extracellular Matrix Bioink for Periodontal Regeneration
Source: Adv Sci (Weinh). 2022 Dec 14;10(5):2205041. doi: 10.1002/advs.202205041 (PMC9929114; doi:10.1002/advs.202205041)
Supplement: Supplementary file 1 — Supporting Information [file ADVS-10-2205041-s001.pdf]

ADVANCED SCIENCE
Open Access

Supporting Information

for *Adv. Sci.*, DOI 10.1002/advs.202205041

A 3D-Bioprinted Functional Module Based on Decellularized Extracellular Matrix Bioink for Periodontal Regeneration

Xueting Yang, Yue Ma, Xiuting Wang, Shengmeng Yuan, Fangjun Huo, Genzheng Yi, Jingyi Zhang, Bo Yang and Weidong Tian**

Supporting Information

A 3D-bioprinted functional module based on decellularized extracellular matrix bioink for periodontal regeneration

Xueting Yang[1-4]†, Yue Ma[1-3]†, Xiuting Wang[1-4], Shengmeng Yuan[1-4], Fangjun Huo[1-3], Genzheng Yi[1-4], Jingyi Zhang[5], Bo Yang[1-4], Weidong Tian[1-4]***

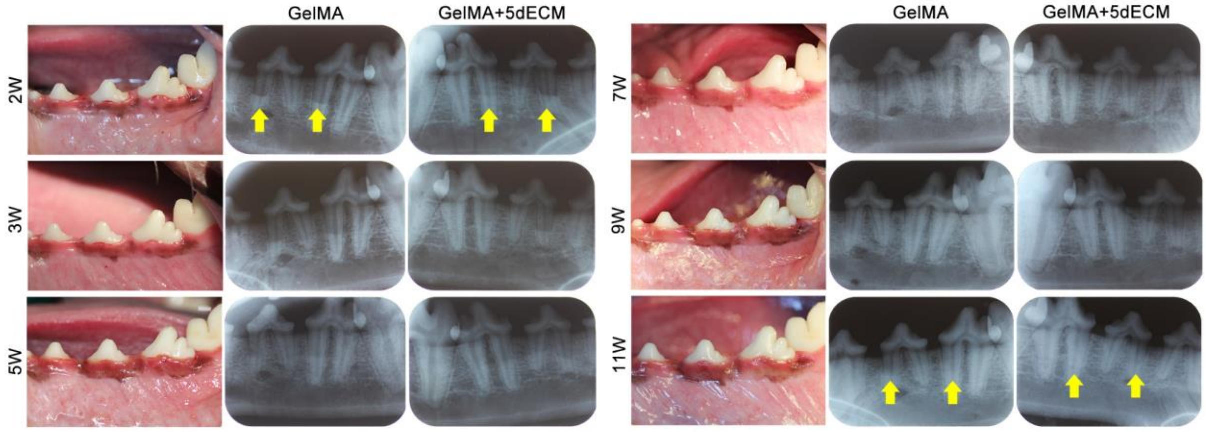

Figure S1. Postoperative follow-up within 3 months of transplantation (yellow arrow: defect area).

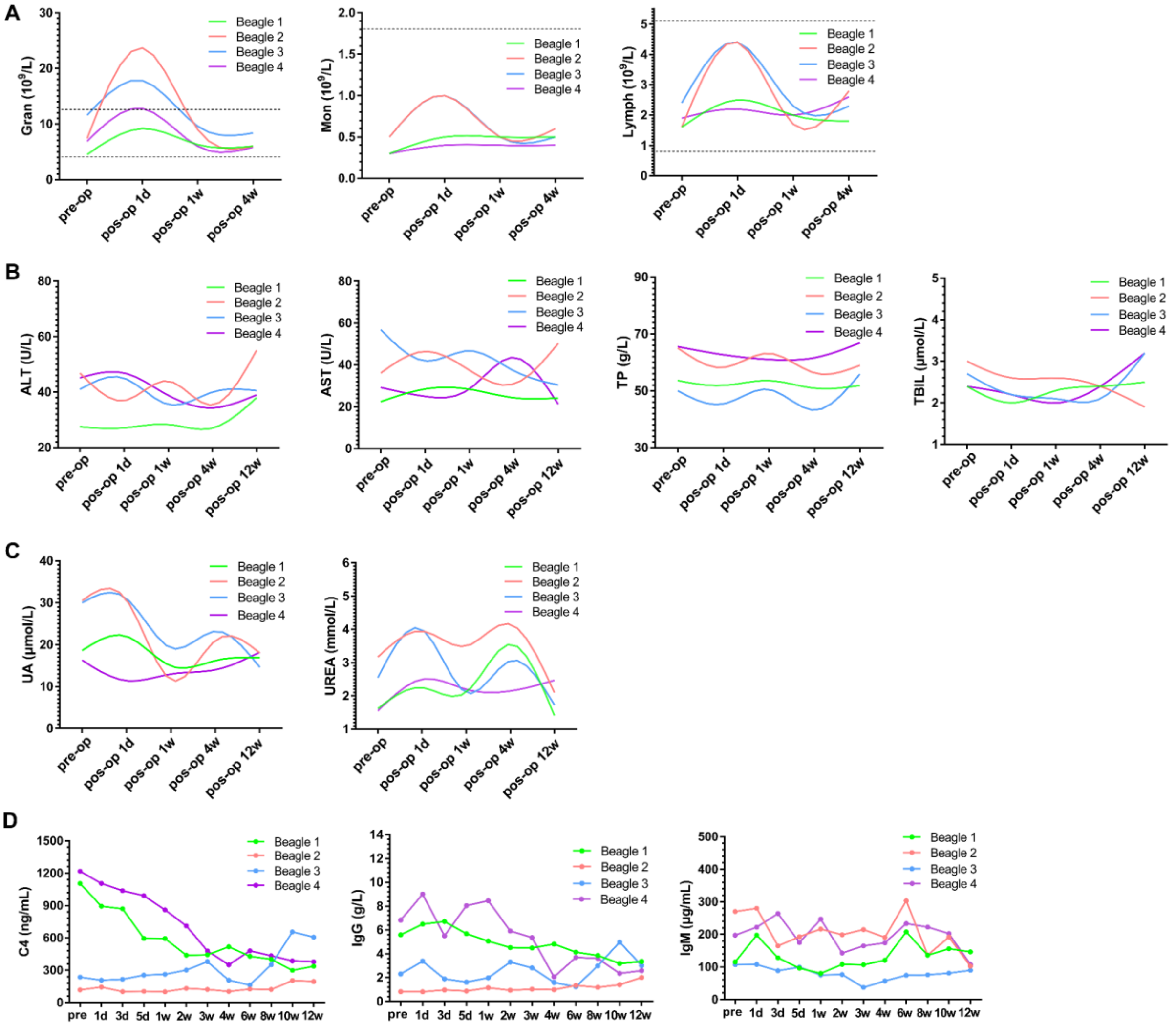

Figure S2. Dynamic monitoring of inflammatory, biochemical and immune indexes in host beagles after module transplantation. (A) The content of granulocytes, lymphocytes, and monocytes in routine blood tests (black dotted lines: the normal range). (B) The content of alanine aminotransferase (ALT), aspartate aminotransferase (AST), total protein (TP) and total bilirubin (TBIL) in liver function analysis. (C) The content of urea and uric acid (UA) in kidney function indicators. (D) The content of immunological indexes in the serum, including C4, IgG and IgM.

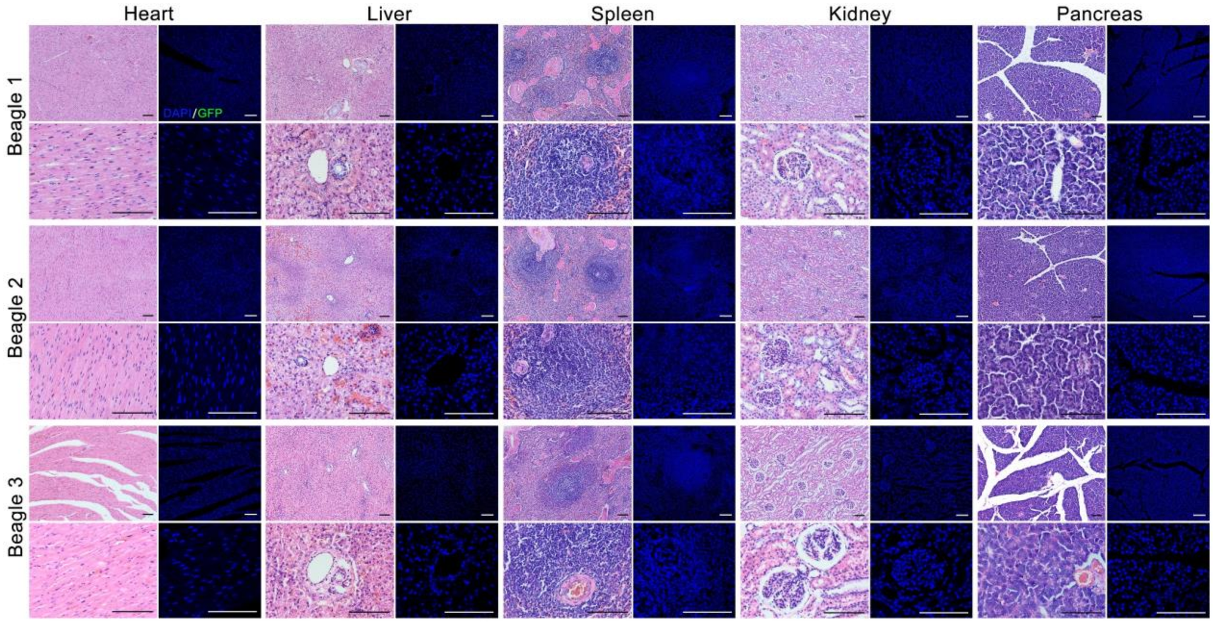

Figure S3. Safety evaluation of transplanted modules. Neither organic lesions in H&E staining nor GFP-positive cells in IF staining were found in any of the tissues. Scale bars = 100 μm.

Table S1. The pressure for extruding out four hydrogels from a 260 μm needle.

	GelMA	GelMA +2.5dECM	GelMA +5dECM	GelMA +10dECM
Minimum pressure	70 kPa	90 kPa	100 kPa	130 kPa
Stable pressure	90 kPa	130 kPa	150 kPa	220 kPa

Table S2. Oligonucleotide primer sequences.

Target cDNA	Primer sequence (5 ' -- 3 ')	Target cDNA	Primer sequence (5 ' -- 3 ')
Runx2	F CTTTACTTACACCCCGCCAGTC<br>R AGAGATATGGAGTGCTGCTGGTC	IL-1β	F ATCTCGCAGCAGCACATCAA<br>R ATGGGAACGTCACACACCAG
ALP	F TAAGGACATCGCCTACCAGCTC<br>R TCTTCCAGGTGTCAACGAGGT	IL-6	F CTCTGGGAAATCGTGGAAAT<br>R CCAGTTTGGTAGCATCCATC
OPN	F CAGTTGTCCCCACAGTAGACAC<br>R GTGATGTCCTCGTCTGTAGCATC	MCP	F CCACTCACCTGCTGCTACTCA<br>R TGGTGATCCTCTTGTAGCTCTCC
COL-1	F AACATGGAGACTGGTGAGACCT<br>R CGCCATACTCGAACTGGAATC	TNF-α	F GTGCCAGCCGATGGGTTGTAC<br>R TGACGGCAGAGAGGAGGTTGAC
DSPP	F CGACATAGGTCACAATGAGGATGTCG<br>R TTGCTTCCAGCTACTTGAGGTC	h-GAPDH	F CTTTGGTATCGTGGAAGGACTC<br>R GTAGAGGCAGGGATGATGTTCT
Periostin	F CACTCTTTGCTCCCACCAATA<br>R ATTTCCTTCCAGCGTCTCAA	m-GAPDH	F AAGAAGGTGGTGAAGCAGGCATC<br>R CGGCATCGAAGGTGGAAGAGTG
Laminin-β1	F ATTCCAACCAGCAGCCGATGTG<br>R GCCGAGGAAGGACGACATATCTTG		
